# A Rare Occurrence of Primary Gastric Ewing Sarcoma

**DOI:** 10.14309/crj.0000000000001494

**Published:** 2024-09-14

**Authors:** Fariha Hasan, Alexander Garcia, Avneet Singh, Natalie Morris, Kathy Williams, Gord Guo Zhu, Adib Chaaya

**Affiliations:** 1 Department of Internal Medicine, Cooper University Hospital, Camden, NJ; 2 Cooper Medical School of Rowan University, Camden, NJ; 3 Department of Gastroenterology, Cooper University Hospital, Camden, NJ; 4 Department of Pathology, Cooper University Hospital, Camden, NJ

**Keywords:** Extraskeletal Ewing sarcoma, primary gastric sarcoma, malignancy

## Abstract

Ewing sarcoma (ES) is a rare malignancy that typically occurs within the skeletal system but can also develop extraskeletally. Extraskeletal ES typically presents paraspinally, in the limbs, and retroperitoneum. Rarely, it presents as a primary gastric ES. To our knowledge, there are only 13 reports of primary gastric ES, none of which originated in the cardia of the stomach. Increased identification of how extraskeletal ES, specifically primary gastric ES, presents and characterized is crucial for future treatment development and accurate prognosis. We present the case of a 36-year-old man with hematemesis, ultimately found to have primary gastric ES in the cardia.

## INTRODUCTION

Ewing sarcoma (ES) is a malignancy derived from mesenchymal cells.^1^ ES is part of a family of tumors, which include the typical ES of the skeletal system, extraskeletal ES (EES), Askin tumors, and primitive neuroectodermal tumors.^1^ These tumors are distinguished by translocations, resulting in fusion genes. Approximately 85% of cases have a (11:22)(q24;q12) translocation, resulting in an EWS-FLI-1 fusion gene.^1^ The remaining 15% of cases result in an ES-electroretinographic (ERG) fusion gene.^1^ ES is a very rare tumor and is typically found in the pelvis, axial skeleton bones, and femur.^2^ The skeleton is where 80%–85% of ES occur.^3^ Even more rarely, ES can be found extraskeletally. EES is most commonly found paraspinally, in the limbs, and in the retroperitoneum.^2^ EES comprises only 15%–20% of ES tumors.^3^ It is extremely rare for EES to be of primary gastric origin, with only 13 cases reported to date, none of which were located in the cardia.^4^ We present a case of a 36-year-old man who presented initially with a single episode of hematemesis, ultimately found to have a primary gastric ES.

## CASE REPORT

A 36-year-old man with no significant medical history presented to an outside hospital with one episode of hematemesis with clots. He subsequently underwent an esophagogastroduodenoscopy (EGD), which revealed severe esophagitis with an exposed vessel and was given pantoprazole. A repeat EGD was scheduled 4 months later, which found a 2-cm gastric polyp, with biopsy showing hyperplastic polyp without evidence of dysplasia. Six months later, the patient presented again to an outside hospital with fatigue and dyspnea on exertion. He was found to be tachycardic and anemic with hemoglobin of 4.5. A repeat EGD was performed, and the previously visualized gastric polyp now appeared ulcerated, measuring 4 cm and extending to the gastroesophageal junction. This broad-based and pedunculated polyp was not biopsied due to the risk of large feeding vessels.

**Figure 1.**
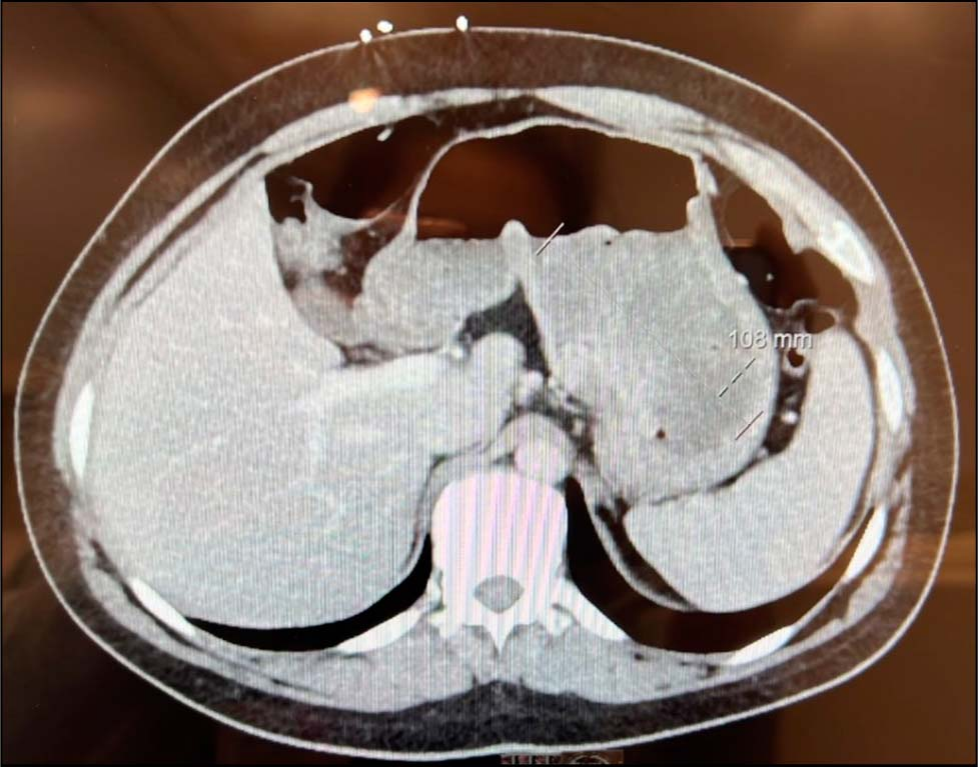
Computed tomography scan of the abdomen with contrast reveals a polypoid mass along the gastroesophageal junction, moderate diffuse gastric wall thickening of the fundus, and proximal body of the stomach.

**Figure 2.**
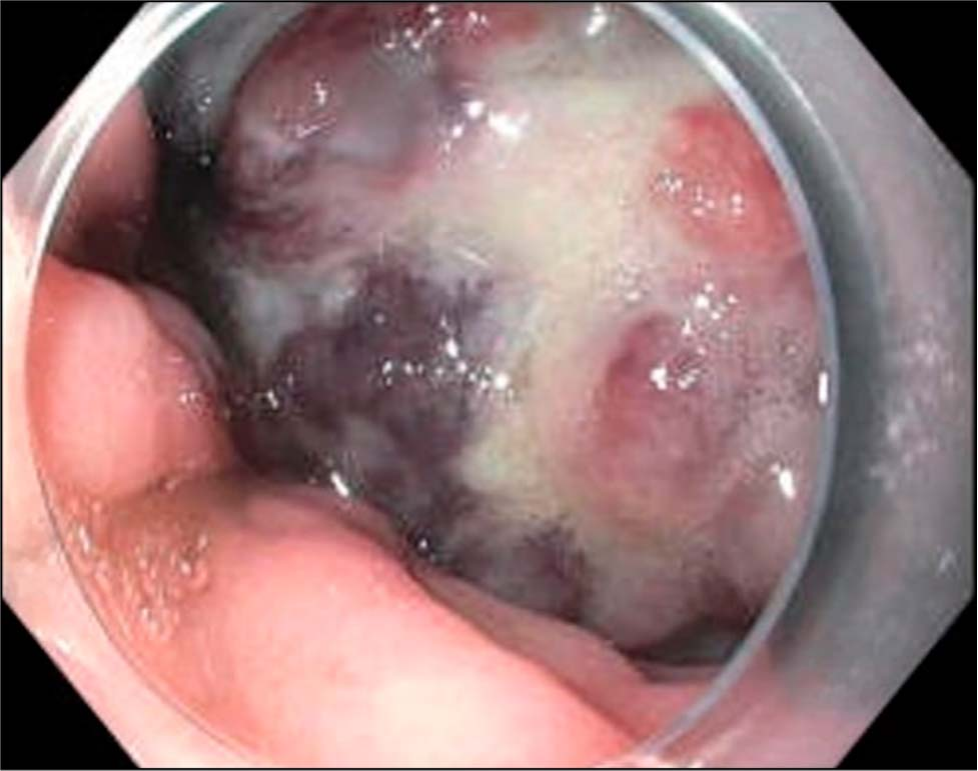
Esophagogastroduodenoscopy revealing a polypoid mass in the cardia of the stomach.

**Figure 3.**
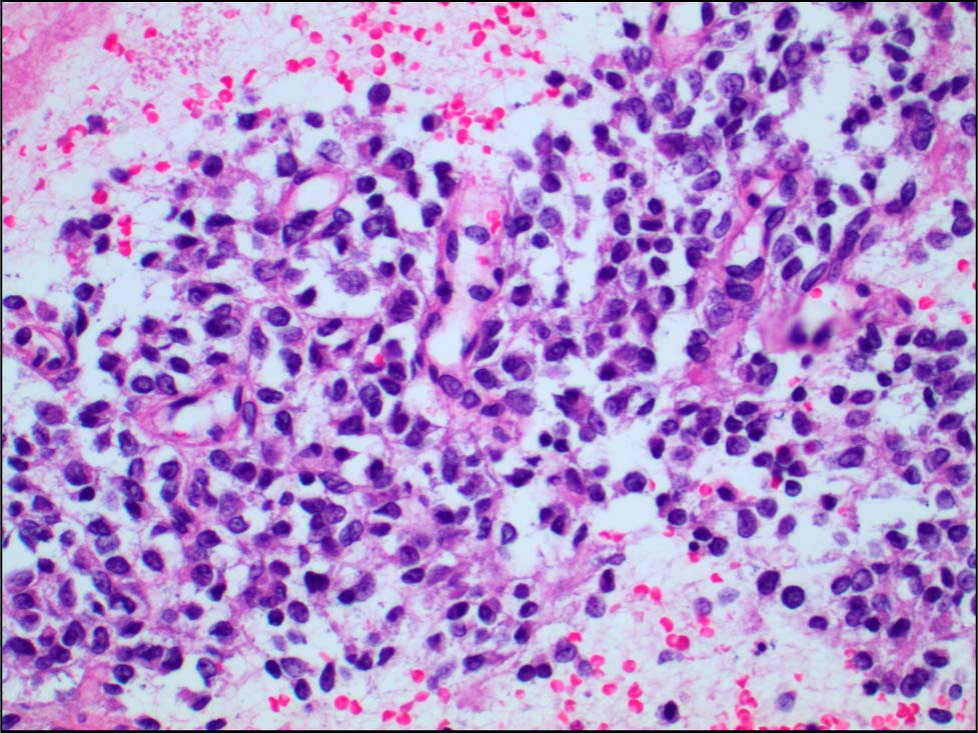
20× magnification, hematoxylin and eosin-stained fine-needle aspiration of gastric mass.

**Figure 4.**
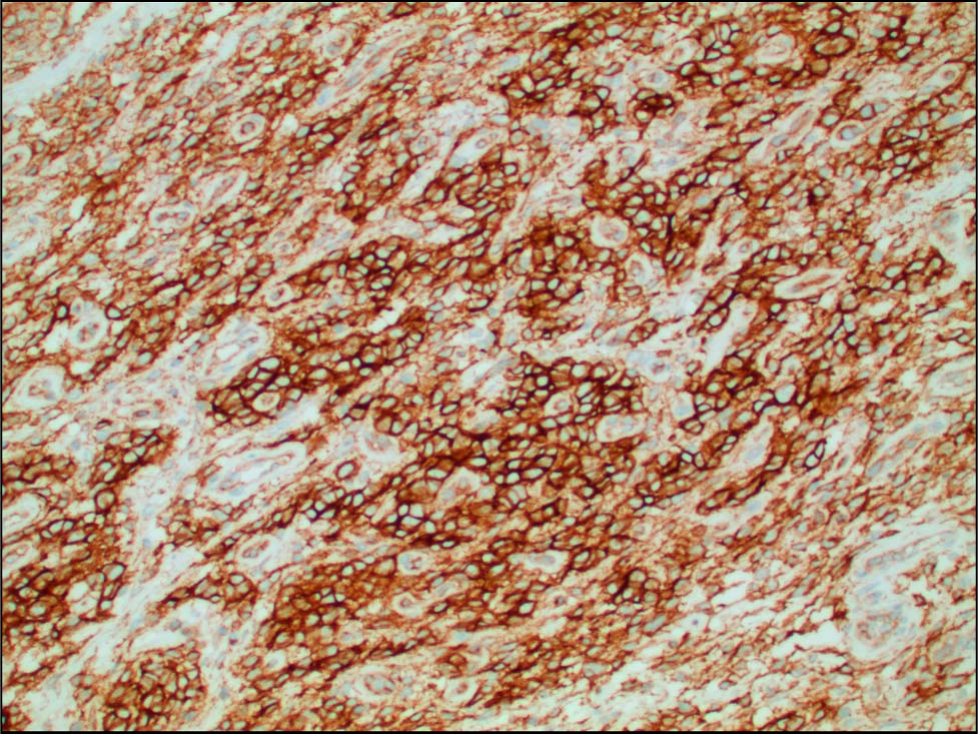
20× magnification, CD99 staining of polypoid gastroesophageal junction mass confirming Ewing sarcoma.

**Figure 5.**
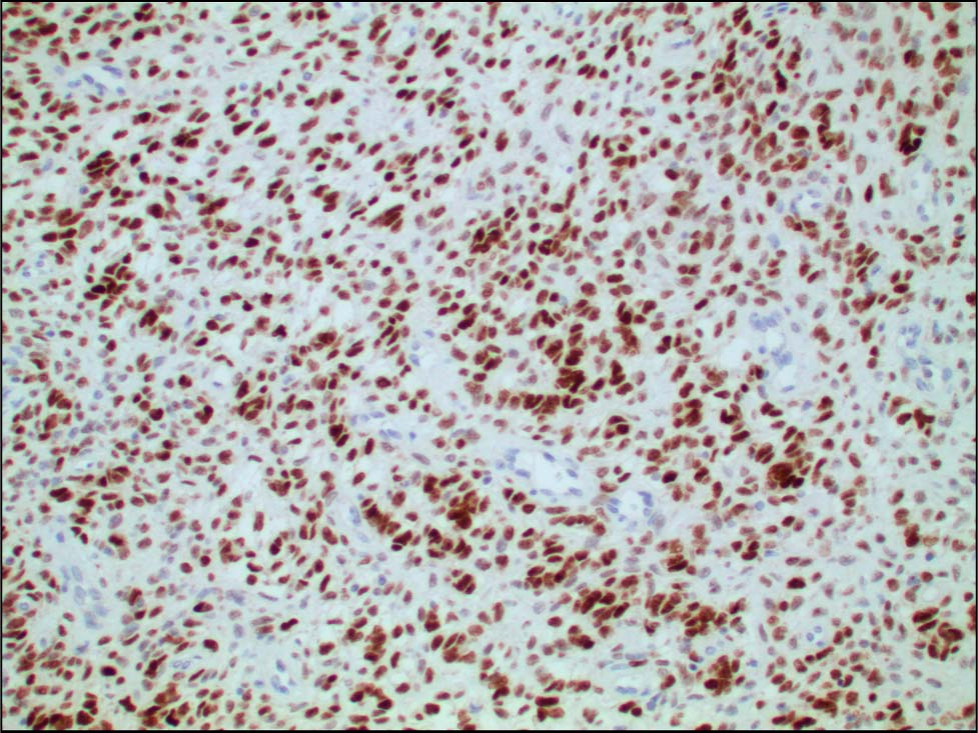
20× magnification, NKX2.2-positive staining of polypoid gastroesophageal junction mass confirming Ewing sarcoma.

**Figure 6.**
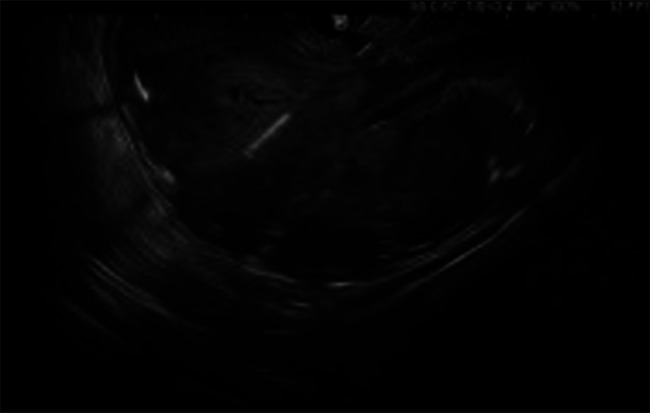
Endoscopic ultrasound showing hypoechoic and heterogeneous pedunculated mass in the cardia, fundus, and body of the stomach, measuring 100 mm by 100 mm in maximal cross-sectional diameter, with well-defined endosonographic borders.

A computed tomography performed at the same time showed a polypoid mass along the gastroesophageal junction, moderate diffuse gastric wall thickening of the fundus, and proximal body of the stomach (Figure 1). A repeat EGD was again performed a month later. It showed a 10-cm mass in the cardia, which extended into the esophagus (Figure 2). A fine-needle aspiration and biopsy of gastric mass were performed (Figure 3). The results were suggestive of a mesenchymal neoplasm, suspicious for sarcoma, with poor differentiation and a Ki-67 of 35%. Staining was positive for vimentin, CD99 (Figure 4), CD56, and CD10. Additional testing confirmed a diagnosis of ES with gene fusion (EWSR1::ERG) and NKX2.2 positivity (Figure 5). It was determined that the mass could not be removed endoscopically, and the patient was therefore transferred to our institution for evaluation by the surgical oncology team. Repeat EGD with endoscopic ultrasound (Figure 6) and fine-needle aspiration were performed. He was ultimately diagnosed with primary gastric ES IIB (T2, N0, M0, and G3). Positron emission tomography scan done for staging revealed no evidence of metastatic disease. He pursued treatment with chemotherapy with a 17-cycle regimen of vincristine, adriamycin, cyclophosphamide, ifosfamide, and etoposide, with a goal of cure. Ultimately, the patient underwent resection of ES, total gastrectomy, esophagojejunostomy, and jejunostomy tube placement. He is currently on cycle 10 of his chemotherapy regimen and is tolerating it well.

## DISCUSSION

The ES family of tumors includes skeletal ES (SES), EES, primitive neuroectodermal tumors, and Askin tumors.^3^ These tumors are derived from mesenchymal progenitor cells and are considered malignant.^3^ While their derivation is alike, it is important to recognize the differences among the 4 tumors. EES is a rare form of malignancy and comprises only 15%–20% of all tumors in the ES family.^3^ Among EES, certain tumor locations are less common than others. One of the most rarest locations for a primary tumor is in the stomach.^3^ A recent study by Shu et al found only 13 cases of primary gastric ES in the literature, including their own case report.^4^ The rarity of this type of EES makes clear the necessity of reporting additional cases of primary gastric ES. Currently, it is recommended to treat EES as same as SES is treated.^5^ However, due to the rarity of EES and especially primary gastric EES, it is not fully elucidated whether this approach is the most beneficial. As highlighted by Shu et al, it is thus critical to acknowledge new case presentations and the treatment regimen received by these rare patients with EES.^4^

Of the 13 cases compiled by Shu et al, none had a location of the cardia of the stomach.^4^ In addition, only 15% of ES cases have a EWSR1::ERG fusion gene, as presented in this case.^1^ Of the few published cases, Yu et al found that primary gastric ES was more commonly found in female patients than male patients with nonspecific symptoms at presentation and a tumor larger than 5 cm.^2^ Our patient contradicts several of these findings. First, he was a male patient. In addition, while his symptoms were not pathognomonic for primary gastric ES, they highly suggested a gastrointestinal location as his initial symptoms included hematemesis. Similarly, his initial presentation was with a 2-cm mass, and while considered benign initially, that same mass is what ultimately was found to be EES. To our knowledge, our patient is also the first reported case of a primary gastric ES originating in the cardia of the stomach. These findings emphasize the high variability in primary gastric ES presentation and importance of reporting and recognizing cases.

The importance of developing knowledge on EES and all its manifestations is well highlighted by Jiang et al that showed differences between EES and SES in terms of survival, with EES having worse survival.^6^ It is unclear why this underscores the importance of continued reporting of EES cases, so further studies can investigate the metastatic pattern and treatment response of EES in comparison with SES.^6^ With increased recognition and reporting, more patterns can be recognized in the future, so early diagnosis and treatment can occur.

## DISCLOSURES

Author contributions: F. Hasan: involved in the conception, acquisition, analysis, and interpretation of data for the manuscript; drafting and reviewing the manuscript; final approval of the version to be published; and agreeing to be accountable for all aspects of the manuscript in question. A. Garcia, A. Singh, N. Morris, GG Zhu: involved in the analysis and interpretation of data for the manuscript; drafting and reviewing the manuscript; final approval of the version to be published; and agreeing to be accountable for all aspects of the manuscript. K. Williams and A. Chaaya: involved in the conception, acquisition, analysis, and interpretation of data; reviewing the manuscript; final approval of the version to be published; and agreeing to be accountable for all aspects of the manuscript. A. Chaaya is the article guarantor.

Financial disclosure: None to report.

Informed consent was obtained for this case report.
